# Factors affecting livelihood choices among the brick workers in Nepal

**DOI:** 10.3389/fsoc.2025.1501129

**Published:** 2025-07-07

**Authors:** Sugat B. Bajracharya, Kamala Gurung, Simran Silpakar

**Affiliations:** International Centre for Integrated Mountain Development, Kathmandu, Nepal

**Keywords:** sustainable livelihood, brick workers, livelihood diversification, informal workers, Nepal

## Abstract

The livelihood choices of the brick workers in their source villages in Rolpa, Salyan and Dang are very limited. Livelihood diversification has been a primary choice for the brick workers due to the seasonality of the brick sector. The factors affecting livelihood choices have been assessed using the sustainable livelihood framework. Using a multinomial regression model for a sample of 500 households spread across the 3 study districts, we identify the factors that determine the choice of main source of livelihood options among the brick workers. We find that an increase in household size, land available for cultivation, livestock count, local market access, and education level is associated with a 2, 15, 5, and 26% higher likelihood of workers selecting agriculture as their main source of income, respectively. In addition to this, external shocks, vulnerability, and changes in trends also impact the availability of livelihood options. Most notably, the COVID-19 pandemic has had a profound impact on livelihood choices with a shift from brick work towards agriculture related work because of the lockdowns in effect at the time of the study. Our findings also show that there is a need for enhancement of capacity of brick workers to sustain their livelihoods by improving their ability to diversify their income sources. Therefore, strengthening the income generation options of the brick workers in the source villages is key to enhance livelihood resilience in the long term.

## Introduction

**Figure 1:**
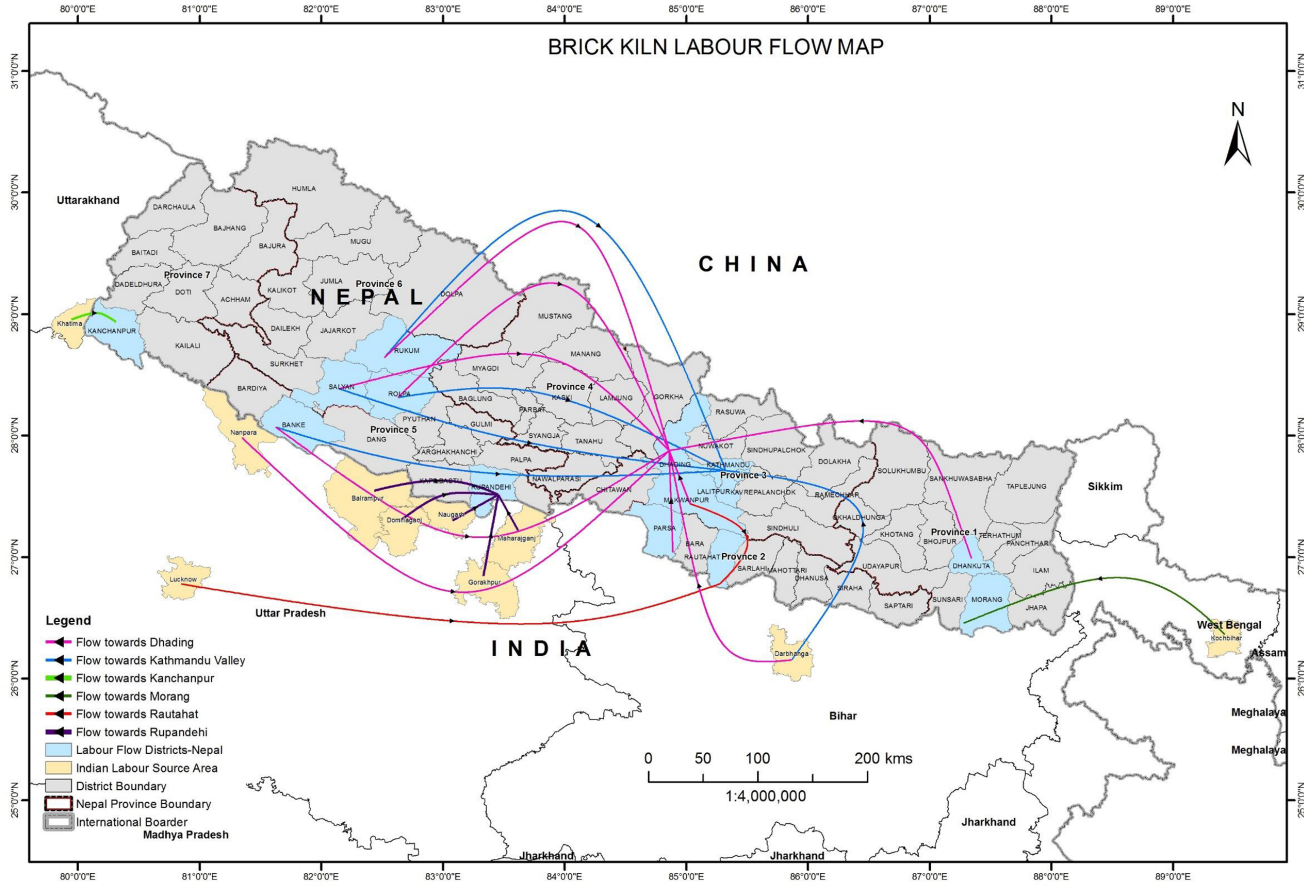
Labor flow into brick industries in Nepal. Source: Figure prepared by Sunil Thapa (ICIMOD).

The brick industry in Nepal is a multimillion-dollar industry providing employment to about 300,000 male and female workers as of 2019 (Sharma et al., 2019). It is estimated that 1,294 brick kilns were functional in Nepal as per the reported figures in 2017 by Global Fairness Initiative (2017). It is a seasonal industry belonging to the informal sector that employs many transient workers from different parts of the country and neighboring country, India as illustrated in Figure 1 (Sharma et al., 2019). The brick industries predominantly comprise migrant workers from marginalized and vulnerable social groups. An ILO report on the brick workers in Nepal shows that most of the workers hail from 6 districts in Nepal—Rolpa, Rautahat, Dang, Kailali, Sarlahi, and Salyan (ILO, UNICEF, and CBS, 2020).

Owing to the seasonal nature of the sector, most of the brick workers are involved in more than one source of employment to support their household livelihoods. Labour migration is a key livelihood strategy in this regard, constituting an integral part of livelihoods that the workers’ households rely on to deal with everyday struggles and mitigate various economic, environmental, and social risks (Olsson et al., 2014; Gioli and Thapa, 2019; Maharjan et al., 2020). Apart from migrating to work in the brick kilns, the workers are also involved in other non-farm labour like construction of roads, houses, labour work, etc. In addition to this, farm labour has also contributed to their livelihood. The diversification of livelihood is a primary option for many brick workers as the six-month seasonal work in the brick kilns often fails to fulfil household need requiring other livelihood sources to complement the income from brick work.

Livelihood dynamics are influenced by multiple climatic or non-climatic driving factors which shape opportunities and decision making (Olsson et al., 2014; Gioli and Thapa, 2019). The climatic factors can include floods, drought, landslides, etc. Non-climatic factors can include economic, social, demographic, technological factors, etc. The key determinants of livelihood and capacity to adapt to climatic and non-climatic changes are livelihood assets and capabilities. In addition to this, the livelihood resources at the disposal of the workers influences their livelihood choices (Gioli and Thapa, 2019).

## Conceptual framework and basic research questions

The study derives its conceptual understanding from the sustainable livelihood framework.

**Figure 2:**
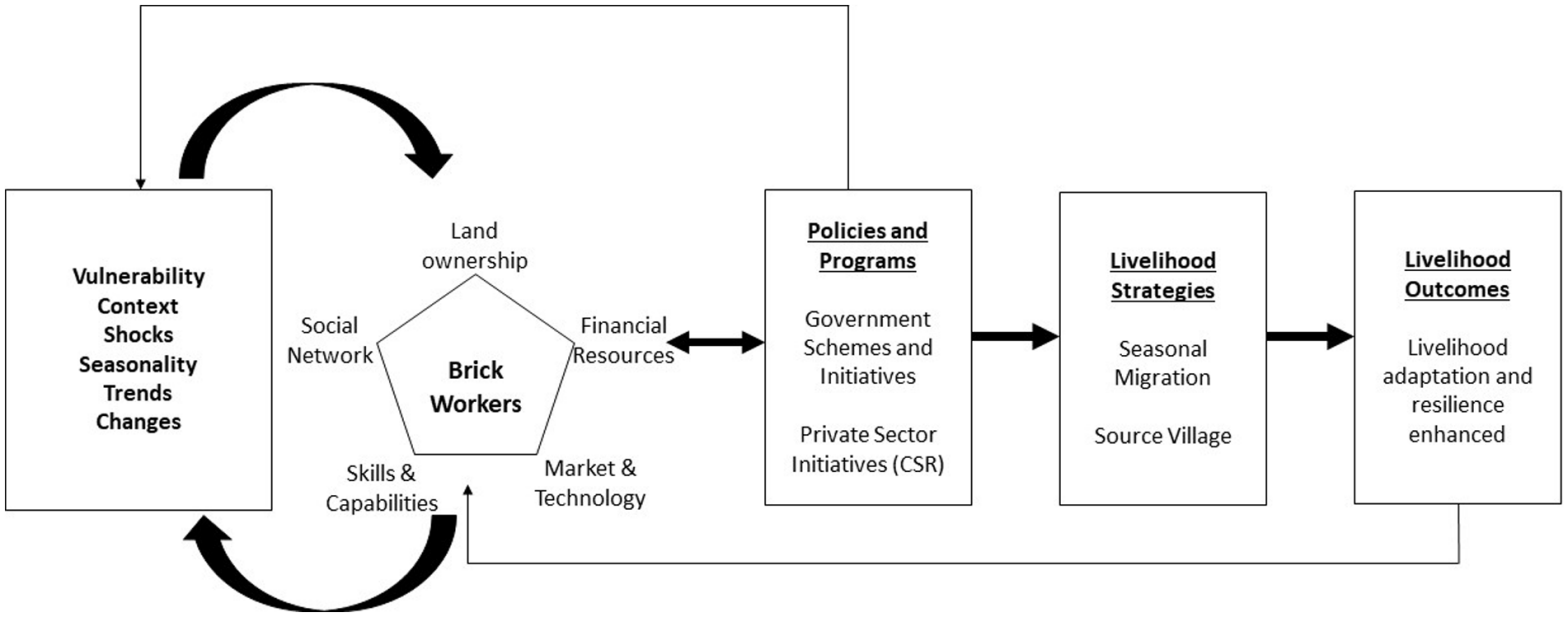
Conceptual framework of sustainable livelihood.

The framework provides a schematic overview of the trajectory of utilizing combination of livelihood resources at their disposal—that can be both tangible and intangible in nature which results in an ability to pursue livelihood strategies to achieve the sustainable livelihood outcomes. It has been specifically referred to understand the factors that determine the livelihood choices (Scoones, 1998). These have been broadly termed as ‘capitals’. There are four types of capitals that have been taken into consideration namely: natural, financial, human, and social capital. The natural capital consists of land that is available for cultivation and number of livestock owned. Financial capital refers to the existence of a financial institution in the village. Physical capital refers to access to local markets (in terms of road infrastructure and market structures). Human capital includes the education of the respondent, skills training (if received) and the availability of household members who can contribute towards household income. Finally, social capital assesses the participation of the respondents in a social and community-based organization, i.e., by collecting information on whether they are a member of such organizations or not (see Figure 2).

We adapted and applied this framework relating it to the specific context of brick workers symbolizing different capitals through its relevant variables. The brick workers, by nature, engage in diverse livelihood options because of the seasonality of their profession which provides employment only for 6 months of a year. For the rest of the year, they seek other means of employment. This livelihood strategy exposes brick workers to vulnerabilities due to the absence or limited livelihood options. Most brick workers come from disadvantaged, illiterate, and vulnerable communities, with limited options for diversifying their livelihoods, making them highly susceptible to exploitation (ILO, UNICEF, and CBS, 2020). The research emphasizes the significance of main sources of livelihood that serve as the bread and butter of the household—the absence of which can have serious consequences for the household’s food and livelihood security. Hence, this research broadly seeks to investigate the determining factors that affect the choice of the main source of livelihood of the brick workers. In addition to this, we delve into a case of two lockdowns during the COVID-19 pandemic to observe the livelihood adaptation strategies adopted by the brick workers in terms of diversifying their incomes through different sources of income to absorb this unforeseen external shock.

## Methodology

### Study area

The study sites have been carefully selected to enable meaningful assessment of the brick workers and their livelihood choices. The source districts (the districts where the brick workers originate from) were chosen among the ones where the highest number of brick workers come from. Subsequently, the districts Rolpa, Dang, and Salyan were selected as study districts as they are among the top six source districts in Nepal according to the report on employment relationship survey in the brick industry in Nepal produced jointly by ILO, UNICEF, and CBS. The report pointed that out of 56,300 workers they surveyed, Rolpa accounted for about 12.6% of the workers, with Dang coming in next at 8.6% of workers and Salyan standing at 5.8%. In addition to the high frequency of brick workers originating from these study sites, they are also a hotspot for the most vulnerable and marginalized groups of people from under-privileged ethnic groups (ILO, UNICEF, and CBS, 2020). Moreover, the remoteness and the difficult terrain of the source villages in the study sites makes sustaining livelihoods a big challenge. As the villages are in hard-to-reach terrains, many are unreachable by motorable roads, making daily life difficult in the villages. Additionally, they are also in a dire need of livelihood projects that have the capacity to sustain their livelihoods for the whole year.

**Figure 3:**
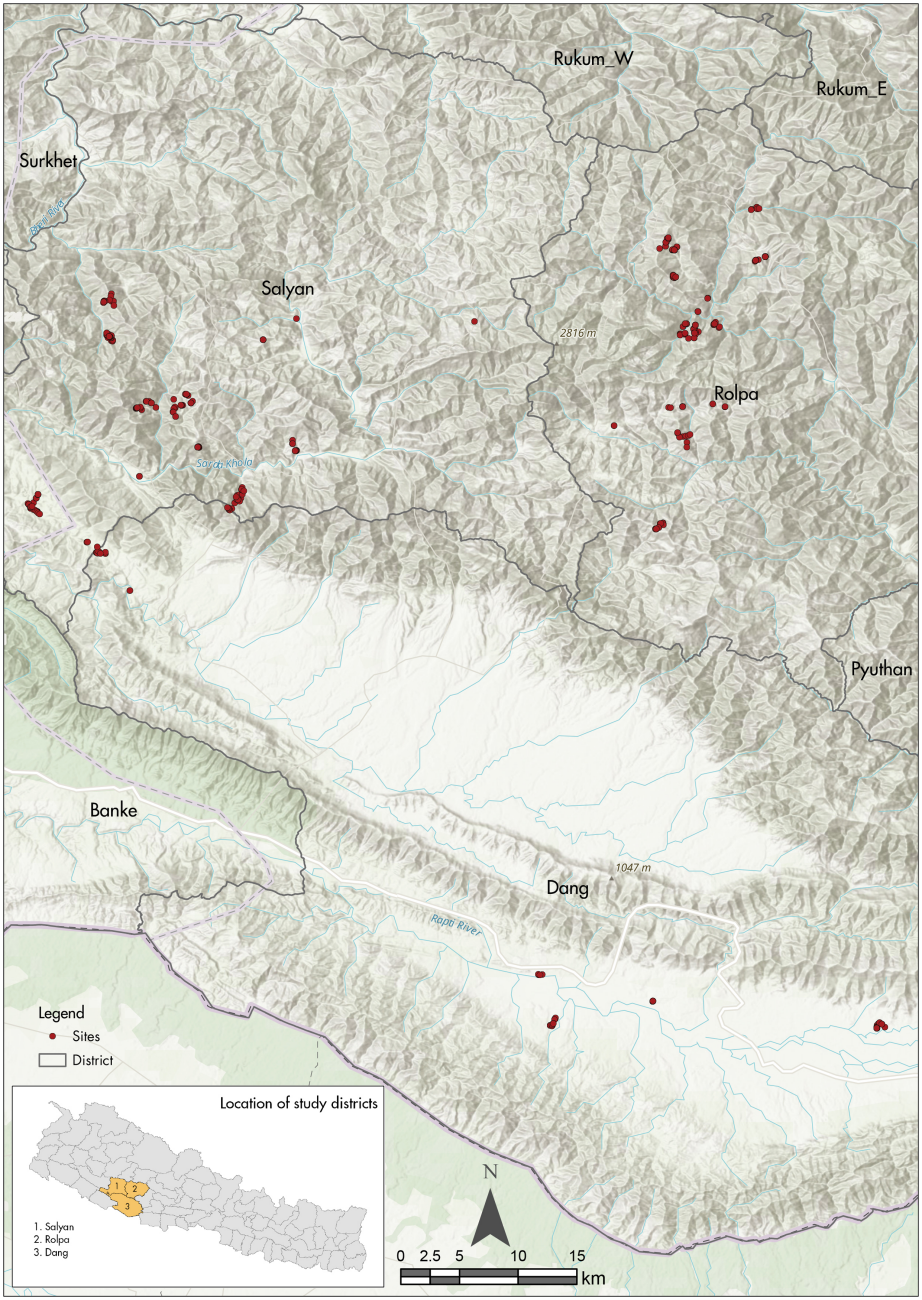
Map of the study area in Rolpa, Salyan and Dang. Source: Map prepared by Gauri Shankar Dangol (ICIMOD).

In the study area of Dang and Salyan districts, there is a high tendency for temporary labor out-migration within Nepal and to India for seasonal work. Even though the agricultural engagement is high, it is limited to subsistence agriculture, contributing very little to the household income (Shrestha et al., 2021). In study sites of both districts, the leading source of income is seasonal brickwork followed by remittance and other wage labor. As is the case for the two study districts, the study sites of Rolpa district also have high seasonal migration for brickwork and labor work in India. People are engaged in subsistence agriculture and are highly dependent on remittances (Pokhrel, 2019). In all the study areas, the women tend to be de-facto household heads because of the absence of males who out migrate to other regions in Nepal or India for labor work. Agriculture land in the study sites is also highly fragmented with small parcels of land ownership among the brick workers in their villages which has discouraged them to carry out agriculture as a major income generation source (see Figure 3).

### Sampling strategy

The sample size of 471 was determined from the Cochran’s method (Cochran, 1977). In the sample estimation, the values of the estimate of variance (0.50), confidence level (95%) and margin of error (0.05) were used. However, to account for possible attrition of respondents, the sample size was rounded off to 500. In the second step, following the method of probability proportionate to size (PPS), a sample of 500 households were distributed across 3 study districts. As there was a lack of data relating to the number of brick workers coming from these districts, the sampling process for the study was determined based on expert and stakeholder consultations regarding the estimated number of population and households that have been engaged in brick work. Through these consultation meetings, it was observed that at least half of the population in Rolpa have at some point involved in brick kilns across the country. Similarly, about two fifths of the population from Salyan were estimated to be involved in brick work with about 10% of the population believed to be working from Dang district. This process can be illustrated from Table 1.

**Table 1:** Targeted households in different districts.

District	Estimated proportion of brick workers from district (*pr*)	Population (as per census 2011) (*P*)	Estimated number of brick workers (*N_i_*) (N_i_ = pr * P)	Estimated number of household (H) (*H=N/hh.size*)	Proportion in total (*P_i_*)	Number of sampled households (*S_i_*) (*S_i_ =* 500 × *P_i_*)	Municipalities purposively selected by expert consultations
Rolpa	0.50	224,506	112,253	22,450	0.43	215	Madi, Paribartan, Triveni
Salyan	0.40	242,444	96977.6	19,395	0.37	185	Bangad Kupinde, Kalimati, Tribeni
Dang	0.10	552,583	55258.3	11,051	0.20	100	Babai, Rajpur, Rapti, Lamahi
Total				52,896	–	500	–

The proportion of estimated brick workers were acquired through expert consultations. Population census numbers for 2011 for each district were then used to get the number of brick workers in each district. This estimated number is divided by assumed household size of 5 to get the estimated household number.

### Data collection

The primary data was collected through the application of quantitative and qualitative methods. The participants of this study are engaged or have worked in the brick sector. The data collection was carried out simultaneously in the three districts. The municipalities of these districts were selected as per suggestions from the brick kiln owners and Naikes (Labor contractors and supervisors) during our scoping study to learn more about the source villages of the brick workers. The number of actual surveyed households came to a total of 544 (Dang—111; Rolpa—218; Salyan—215) keeping in mind possible high attrition rate and to maintain buffer sample in such a case. Qualitative data were gathered through 5 Focus Group Discussion (FGDs) (2 each in Rolpa and Dang; 1 in Salyan) and key informant interviews with 10 Naikes (labor contractors/supervisors). The initial field plans to conduct 10 FGDs in the respective municipalities were not possible due to difficulty in gathering people as a result of difficult terrain. To work our way around it, they were gathered making use of community festivals and events. A pretested questionnaire was used for quantitative data collection. The data was collected with the engagement of eight enumerators using Kobo Toolbox. The pretest was carried out in the brick kilns of Dhading among the workers representing the study districts. Verbal consent was acquired from all the participants for the study. This was done as written consent was difficult to obtain due to unwillingness of the workers to draw up a document and sign it legally.

### Data analysis

The data analysis was carried out through both descriptive statistics as well as model specifications. To identify the factors that determine the choice of main source of livelihood options among the brick workers, we devised a multinomial regression model as we investigate 3 broader categories of main source of household income. The model specifications have been adopted from Dinku (2018). The model can be specified in the following way:


$$P_{\mathrm{ij}}=\frac{\exp\;(X_{i}\beta_{j})}{\sum_{j=1}^{J}\exp(X_{i}\beta_{j})},J=(1\ldots 3)$$

Let $P_{\mathrm{ij}}$ denote the probability of a household choosing a given livelihood option as their main source of income, with *j* = 1 if the household indicates agriculture as their main source of income, *j* = 2 if the household indicates wage labour as their main source, and *j* = 3 if they take up other work as their main source.

The factors that determine the main livelihood choice are obtained by the following equation:


$${\mathrm{MLC}}_{i}=\beta_{0}+\beta_{1}X_{i}+\beta_{2}Z_{i}+u_{i}$$

Following the sustainable livelihood framework by Scoones (1998), the model includes variables relating to the ‘capitals’ in the framework denoted by $X_{i}$ which denotes the vector of different variables relating to natural, human, financial and social capital. $Z_{i}$ denotes other control variables that represent individual and household characteristics.

The description of the variables for the analysis are as follows (see Table 2).

**Table 2:** Description of variables.

Variables	Description
Main source of livelihood	1 = Agriculture; 2 = Wage labour; 3 = Other work
Age of respondent	A Continuous variable measured in years
Sex	Sex of household head (Female = 1; Male = 0)
Education level of respondent	Education level of respondent (1 = Illiterate; 2 = Non-formal education; 3 = Primary; 4 = Secondary; 5 = High school; 6 = Above high school)
Household size (working members)	Number of working household members in the family
Land area for cultivation	Cultivable land area in hectares
Livestock count	No. of livestock in the household
Local market access	Access to local markets (in terms of road infrastructure and market structures) (Yes = 1; No = 0)
Social institutional affiliation	Member of any social organization (Yes = 1; No = 0)
Financial institution in village	Financial institution located in the village (Yes = 1; No = 0)
Dalit	Caste classifications (Dalit = 1; Non-dalit = 0)
Skill training	Taken skill training (Yes = 1; No = 0)

## Results and discussion

### Basic characteristics of brick workers

The brick workers in the study areas have demographic and socioeconomic characteristics as shown in Table 3. The average age of the respondents was 31. About 70% of the workers interviewed were male. The educational profile of these workers shows that 60% of them have not completed high school with almost 23% illiterate. Majority of the brick workers (60%) in the study sites primarily belonged to Janajati ethnic group with about 24% belonging to the Dalit group. The average household size contained 5 family members with livestock count averaging to about 3. Average cultivable landholding size came to around 0.08 hectares. For easier interpretations of the results, marginal effects of the change in the predictor variables on the outcome variable have been produced in Table 4.

**Table 3:** Summary statistics of characteristics of brick workers.

Variable	Obs	Mean	Std. Dev.	Min	Max
Age of the respondent	544	31.65441	11.11509	16	72
Sex of the respondent	544	0.702206	0.457709	0	1
Education					
Illiterate	544	0.237132	0.425716	0	1
Informal education	544	0.097427	0.296811	0	1
Education under high school level	544	0.608456	0.488545	0	1
Education high school level and above	544	0.056985	0.232028	0	1
Caste					
Dalit	544	0.248162	0.432344	0	1
Janajati	544	0.615809	0.486851	0	1
Brahmin	544	0.016544	0.127673	0	1
Chettri	544	0.075368	0.264227	0	1
Other ethnicity	544	0.044118	0.205546	0	1
Household size	542	5.359779	1.984619	2	18
Land area for cultivation (in Ha)	542	0.085936	0.192104	0	2.5
Livestock count	541	2.807763	1.221723	1	7
Main source of income					
Agriculture	544	0.246324	0.431266	0	1
Wage labour	544	0.71875	0.450023	0	1
Other	544	0.027574	0.163898	0	1
No. of years of work in brick sector	544	5.130515	4.69821	1	35
Type of brick work					
Molder	544	0.220588	0.415025	0	1
Stacker	544	0.020221	0.140884	0	1
Transporter	544	0.683824	0.465411	0	1
Transporter (truck)	544	0.018382	0.134453	0	1
Other type of brick work	544	0.056985	0.232028	0	1
Availability of local market access in source village	544	0.305147	0.460894	0	1
Social institutional affiliation	539	0.211503	0.408753	0	1
Availability of financial institution in source village	539	0.142857	0.350252	0	1
Skill training	544	0.198529	0.39926	0	1

**Table 4:** Factors affecting the choice of main source of livelihood (marginal effects).

Variables	Agriculture	Non-farm labor	Other work
Age of respondent	0.0024	−0.0046**	0.00211**
Sex (Female = 1, Male = 0)	0.0706*	−0.1430***	0.0724**
Education level of respondent	0.0417***	−0.0584***	0.01675**
Household size (working members)	0.0206*	−0.0207*	−0.00009
Land area for cultivation	0.1506*	−0.1324	−0.0182
Livestock count	0.0554***	−0.0547***	−0.0006
Local market access	0.2672***	−0.2470***	−0.0201
Social institutional affiliation	−0.0297	0.0229	0.0067
Financial institution in village	0.0327	−0.031	−0.0016
Dalit (Dalit = 1, Non-Dalit = 0)	−0.022	0.0253	−0.0031
Skill training	0.0477	−0.0562	0.0085

*** 1%, ** 5%, * 10%.

The main source of income is wage labour, which accounts for about 71% of the workers. Similarly, around 24% of them considered agriculture as their main source of income. Among the workers who stated wage labour as their main source of income, most of them pointed to brick kiln work as their first choice or priority choice of work. The second choice comes in the form of labour work in India, followed by other labour work in Nepal. This shows that brickwork attracts many to stay in the country but due to seasonality in the brick sector, are required to look for other options to sustain their livelihoods. The average number of years of work in the brick sector is observed to be 5 years. Many of the brick workers (68%) worked as brick transporters in the brick kilns with about 22% involved in molding bricks.

### Mobility of brick workers in different livelihood activities

The brick workers are mainly involved in farm, off-farm and non-farm livelihood activities. As observed from field and group discussions, the brick workers in the study areas are usually involved in three different livelihood activities namely: agriculture-based activities (involving livestock, horticulture, farming, etc.) from May to July at their source villages, seasonal migration to India from July to October, and internal migration to brick kilns in the country for the duration of November to May. These have been observed through group discussions and field observations (see Table 5).

**Table 5:** Mobility of brick workers in different livelihood activities.

Sector	Work activities	Jan	Feb	Mar	Apr	May	Jun	July	Aug	Sept	Oct	Nov	Dec
Non-farm	Internal migration to work in the Brick kiln.												
	Seasonal migration to India for non-farm labor work (for, e.g., labor work in Himachal Pradesh, Delhi) (males usually migrate for this purpose).												
Farm	Agriculture-based activities (livestock, horticulture, etc.).												

Source: Field observations and group discussions.

### Factors determining the main source of livelihood

The findings of the multinomial regression model give us insight into different factors that affect the choices of the main source of livelihood for the brick workers that originate from the study districts of Dang, Rolpa, and Salyan. The results show that six factors (that are statistically significant) tend to affect the probability of choosing agriculture as their main source of livelihood (Table 4). These factors are the sex and education level of the respondent, household size (working members), land area available for cultivation, livestock count, and local market access. It is observed that women brick workers compared to men were 7% more likely to choose agriculture as their main source of livelihood. A positive association is observed between human capital enhancement and the choice of agriculture as their main source of income. An increase in one unit in the level of education among the brick workers is associated with a 4% increase in likelihood of choosing agriculture as their main source of household income. Skill training has a positive association with the likelihood of choosing agriculture as main source of income. However, the results are not statistically significant. The number of working members in the family is also positively associated with the choice of agriculture showing a 2% higher likelihood for a one-unit increase in the number of members. This finding is in line with the results obtained by Ahmad et al. (2023) which shows that households that have more members in the working age were more likely to enter farming. The presence of adult labor provides more hands for the farm operations such as fertilizing, weeding, transplanting, harvesting, etc. Similarly, there is also a positive association between natural capital and the choice of agriculture as the main income. A one unit increase in land area available for cultivation and livestock count in the farm is associated with 15 and 5% increase in likelihood of selecting agriculture as livelihood, respectively. This is in line with observations made in Gurung (1987), Herrero et al. (2010), Ahmad and Ma (2020), and Ahmad et al. (2023) wherein mixed-crop livestock production is considered a vital part of livelihood aided by land and livestock ownership. Additionally, households who owned land (family farm) and livestock (buffalo, cattle, sheep, goats, etc.) were significantly less likely to exit from farming (Ahmad et al., 2023). In relation to financial capital, it is also positively associated with the likelihood of choosing agriculture as main income source. Local market access is significantly associated with a 26% increase in likelihood of choosing agriculture as the main source. A large body of studies have investigated into factors influencing farmers’ market participation and their inclination to make it a commercial means of earning (Abate et al., 2022; Alene et al., 2008; Assefa and Getachew, 2023; Haile et al., 2022; Rubhara and Mudhara, 2019). Most have highlighted the significance of access to market in deciding on farming as commercial source of income. The existence of a financial institution in the village is also positively associated with the choice. However, this is not seen to be statistically significant.

Similarly, when we analyze the factors that determine the choice of non-farm labour as the main source of income, there are also six factors that are deemed to be statistically significant. The demographic factors like the age (older folks were less likely to select non-farm labour) and sex of the workers (women were 14% less likely to be choosing non-farm labour) is seen to be significant. With regards to human capital, higher education level and increase in working members in the household were associated with the less likelihood of choosing non-farm labour by 5.8 and 2%, respectively. On the natural capital front, higher livestock count was associated with less likelihood (5%) of choosing non-farm labour. Local market access is significantly associated with 24% decrease in likelihood of choosing non-farm labour as main source.

### Livelihood shifts during and between COVID19 induced lockdowns

Unforeseen external shock is one of the factors that affect the livelihood choices. This has been demonstrated clearly by the labour mobility and livelihood changes that have happened due to the COVID-19 pandemic induced lockdowns. Each of the lockdowns roughly lasted about 5 months—the first lockdown lasted from March/April of 2020 to July/August of 2020 with the second lockdown duration being from April 2021 till August 2021. This shock had triggered movement of the brick workers (in terms of location) as a means of adjusting and adapting to the situation of COVID-19. A similar scenario was observed in India according to the study conducted amongst brick workers by Bhattacharya (2020) in Uttar Pradesh and Rajasthan which showed that movement was observed to cope with the shock triggered by the pandemic and adapt to change in their livelihood (Bhattacharya, 2020).

**Figure 4:**
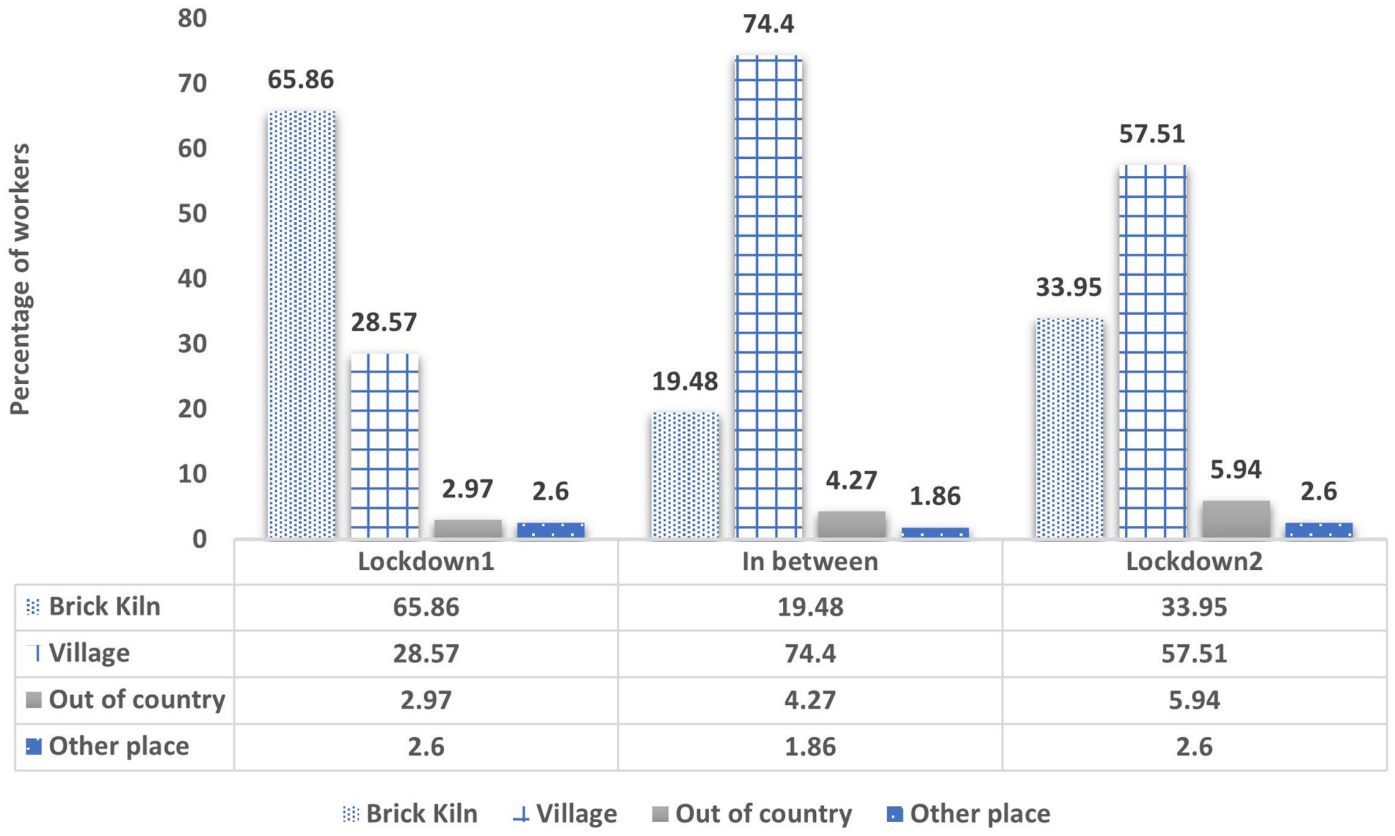
Mobility of brick workers during and between lockdowns.

During the first lockdown, about 65% of the brick workers stayed at their respective brick kilns due to the uncertainty surrounding COVID-19. A high movement of labour specifically took place between the two lockdowns as the brick workers—the majority (about 74%) of them—made their way back to their villages (see Figure 4).

As the second lockdown arrived, about 57% of the brick workers were in their villages with only 33% of the workers staying in the brick kilns. As brick kilns shut down due to the lockdowns, the brick workers pursued their livelihood back in their villages.

**Figure 5:**
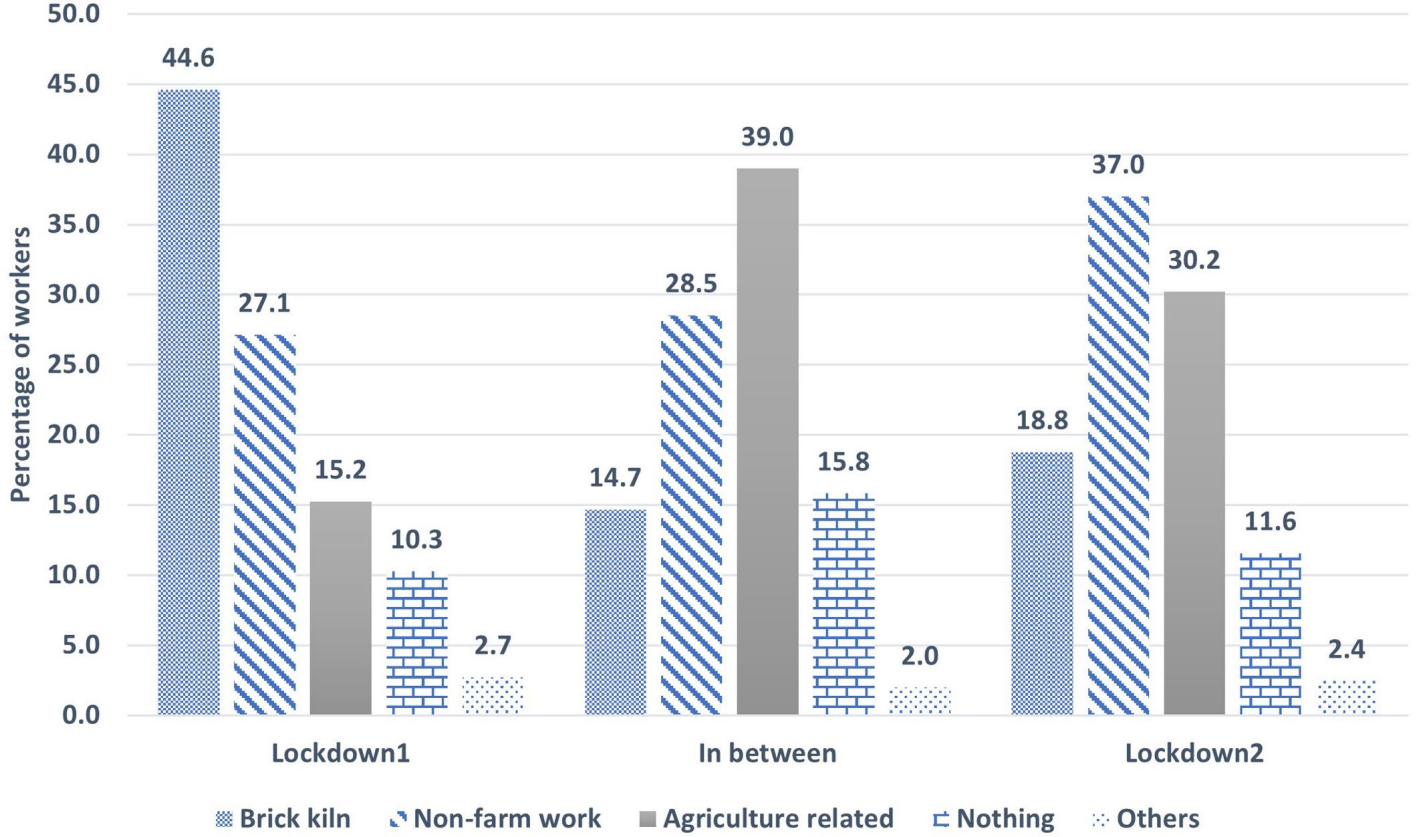
Different livelihood choices of the brick workers during and between lockdowns.

Similarly, we see a shift in livelihood during and between the lockdowns as the workers assess their situations and adapt (illustrated in Figure 5). There are different livelihood options that the brick workers take up as they move from one lockdown to another. During the first lockdown, about 45% are still working in the brick kilns at some capacity. 27% of the workers are engaged in other forms of non-farm work with only 15% engaged in agriculture related work. 10% are out of work and doing nothing at this stage.

In between the lockdowns, we see a shift from brick kiln work to agriculture related work as most of the brick workers are back in their villages. Only 14% of the workers remained working in the brick kilns in the duration between the two lockdowns. 39% of the workers were engaged in agriculture related work during this period. About 28% were engaged in other forms of non-farm work while 15% of the workers were out of work at this stage.

During the second lockdown, we see a balancing out of the livelihood choices taken up by the workers. We see 37% of the workers involved in other non-farm related work with 30% involved in agriculture related work. Only 18% of the workers are seen to be working in the brick kilns in this period with 11% of workers being out of work.

### Discussions on implications of the findings

The livelihood dynamics in the study districts among the brick workers have demonstrated that livelihood diversification has been in practice in one form or other as a primary option due to the seasonality of the brick sector. This means that the brick sector workers have been adept to working multiple jobs to sustain their livelihoods. Majority of the brick workers tend to migrate to India during the off-season (usually from July to Oct). While some are involved in agriculture in their respective villages, only about 24% of the brick workers relied on it as their main source of livelihood. To make matters even more critical, the hilly terrain and low average landholding size of agricultural land that is well below the national average is quite challenging for commercial agricultural ventures.

The capacity of the brick workers to diversify and sustain their livelihoods is lacking in agriculture/livestock farming to make it a viable and main livelihood option. The agriculture and livestock combo or often referred to as mixed agriculture looks to be a worthy proposition which has been taken up by projects like Generating Opportunities in Agriculture and Livestock (GOAL) with financial support from Australian Aid. The project has been promoting vegetable farming and goat farming in Runtigadhi rural municipality of Rolpa. The involvement of the local government in partnership with the implementing partners have been the highlight and main operational modality of the project (AusAID, ADRA, 2022). This needs to be followed up by increased access to cultivable land through lease/renting arrangements that involve farmer co-operatives facilitating the prospect of commercial agriculture.

To make agriculture/livestock farming as a viable livelihood option, there is a clear need for a better access to market. Rolpa, one of the less connected among the study sites selected, requires intervention towards easing market access for the local farmers. Holeri Collection Centre in Holeri, Rolpa had been established in 2011 to facilitate trading of farmers’ produce and maintain a marketplace for agricultural products. However, the center was not operational due to underlying issues relating to its planning, management and operations. It was revived again by USAID funded project Knowledge-based Integrated Sustainable Agriculture and Nutrition (KISAN) in 2014 through an initiative to involve active famers, better management through management trainings, capacity building, etc. The main underlying issue here remains the consistency and sustainability of the operation of the collection center (CEAPRED, 2015).

A true resilience capacity can be observed during the times of crisis. The livelihood diversification options exercised by the brick workers can mostly be categorized into farm and non-farm labor that have been prevalent during the COVID-19 pandemic. The brick workers seem to have adapted by shifting their livelihood options as the pandemic took shape albeit gradually returning to their prominent work in the end. This holds true for many other informal workers in India as per evidence documented by Bhattacharya (2020) from Uttar Pradesh and Rajasthan. Although some of the informal workers in Colombia appeared to leave their former occupations to pursue new ones, many transitioned to a new livelihood for a time before returning to their original jobs (Staupe-Delgado and Díaz Villarreal, 2023).

One key adaptation measure taken by brick workers to cope with COVID-19 is their increased reliance on agriculture in their villages as a source of subsistence income. This points to the importance of establishing a resilient livelihood base that can sustain their livelihoods in times of crisis and shocks. In addition to this, there is also a lack of skillsets to allow for different livelihood options through training and capacity building in the form of vocational programs and courses. These types of labor market programs in the form of skill development trainings are direly needed in this context. Moreover, as it is difficult for informal workers to deal with unforeseen shocks, public works programs that provide employment supports are needed. One example of this can be seen in India under Mahatma Gandhi National Rural Employment Guarantee Act that provides seasonal agriculture employment in rural areas (Handayani and Wening, 2016).

## Conclusion

The livelihood choices of the brick workers in the source villages in Rolpa, Salyan and Dang are very limited. This has led to many migrating to other districts for work—with the primary income source being wage labour in the brick kilns. While agriculture is still a mainstay in the villages, it is not a major source of income for most brick workers and is mostly used for subsistence. This has largely been determined by the resources at their disposal in the form of different capitals available, i.e., natural, financial, human, and social capital. We find that the likelihood of the workers choosing agriculture as a main source of income is determined by a host of factors like household size, land available for cultivation, livestock count, local market access. In addition to this, external shocks, vulnerability, and changes in trends also impact the availability of livelihood options. Most notably, COVID-19 pandemic has had a profound impact on the choices of livelihood with a shift from brick work towards agriculture-related work because of the lockdowns in effect.

The findings show that there is a need to enhance the capacity of brick workers to sustain their livelihoods by improving the agriculture/livestock farming as a viable option. While there have been livelihood projects that have focused on it, more needs to be done in terms of outreach of the programs. Moreover, there is a dire need for establishment of market access mechanism for agricultural products that is operational on a consistent basis. At the same time, a proper livelihood diversification can take place only when the brick workers are adept in other skills required for other jobs. This requires capacity building through vocational trainings that uplift the skill level.

However, there is a need to consider the limitations of the study as the analysis has not explored certain aspects relating to financial access (like informal means of access through family and relatives); livestock count has not included specific livestock type and converted into a livestock unit. Relevant variables have been included as per the brick workers’ context but it could be improved to include informal workers in general.

## Recommendations

Livelihood diversification has been a primary option for the brick workers due to the seasonality of the brick sector. Hence, on the longer-term basis, it is key to enhance their livelihood resilience by strengthening the livelihood options in the source villages. To achieve this, following recommendations can be suggested:

1. *Strengthen the livelihood options in agriculture and livestock*—Boosting agricultural production through better irrigation, seeds production, value added processing, resource efficiency and product marketing can help in creating a reliable source of household income to further support income diversification. There is a need for appropriate programs, investment and interventions geared towards commercializing agriculture where scope exists. Along the same lines, livestock farming provides a possibility for alternative income potential for, e.g., goat farming can be commercialized.
2. *Strengthen the market accessibility in the region*—The findings point towards 26% higher likelihood for the workers to consider agriculture as their main source of income when there is reliable market access. Hence, establishment and enhancement of market mechanism is key towards a better agriculture-led income-generating streams (commercial crop production and livestock rearing).
3. *Establishing financial institutions and providing financial literacy in the villages*—Lack of financial institutions and literacy in the villages have contributed towards lack of investment in income generating activities by the workers. There’s a large scope for microfinance in Nepal’s context to create access to credit, savings, and investment opportunities. Additionally, provision of training on financial literacy is required to build entrepreneurial mindset and capability.
4. *Vocational training*—Vocational training among the brick workers beyond brick industries is required to enhance skills in other work areas that can enhance the livelihood resilience of these workers enabling them to diversify their livelihood options. Providing vocational training in alternative sectors can equip brick workers with new skills to pursue during the off-season. The skills can be tailored to market demand and job and opportunities availability.
5. *Support access to social protection programs*—Ensuring access of brick workers to social security and protection schemes such as unemployment benefits and insurance can help them mitigate and mange risks and shocks such as COVID-19.

## Data Availability

The original contributions presented in the study are included in the article/supplementary material, further inquiries can be directed to the corresponding author.
